# Private Insurance and Mental Health among Older Adults with Multiple Chronic Conditions: A Longitudinal Analysis by Race and Ethnicity

**DOI:** 10.3390/ijerph18052615

**Published:** 2021-03-05

**Authors:** Hankyung Jun, Emma Aguila

**Affiliations:** Sol Price School of Public Policy, University of Southern California (USC), Los Angeles, CA 90007, USA; eaguilav@usc.edu

**Keywords:** multiple chronic conditions, mental health, cognitive health, health insurance, race disparity

## Abstract

Older adults with multiple chronic conditions have a higher risk than those without multiple conditions of developing a mental health condition. Individuals with both physical and mental conditions face many substantial burdens. Many such individuals also belong to racial and ethnic minority groups. Private insurance coverage can reduce the risks of developing mental illnesses by increasing healthcare utilization and reducing psychological stress related to financial hardship. This study examines the association between private insurance and mental health (i.e., depressive symptoms and cognitive impairment) among older adults in the United States with multiple chronic conditions by race and ethnicity. We apply a multivariate logistic model with individual fixed-effects to 12 waves of the Health and Retirement Study. Among adults with multiple chronic conditions in late middle age nearing entry to Medicare and of all racial and ethnic groups, those without private insurance have a stronger probability of having depressive symptoms. Private insurance and Medicare can mediate the risk of cognitive impairment among non-Hispanic Whites with multiple chronic conditions and among Blacks regardless of the number of chronic conditions. Our study has implications for policies aiming to reduce disparities among individuals coping with multiple chronic conditions.

## 1. Introduction

One in four U.S. adults have multiple chronic conditions (MCC) [1,2]. Older adults with MCC have higher risks of developing a mental health condition. Patients with more health conditions tend to experience higher levels of depression [3,4,5]. Dementia and cognitive impairment are associated with cardiovascular disease [6], hypertension [7], obesity [8,9], diabetes [10], and stroke [11]. Previous research shows that cognitive impairment manifests more among individuals older than 78 years of age [12]. This suggests that those at older ages with MCC are also more likely to experience cognitive impairment.

Poor mental health itself can significantly reduce the well-being of older adults. Having a mental illness in addition to chronic conditions can adversely affect quality of life by increasing medical costs, use of emergency services [13,14], and risk of other illnesses [15], as well as by impeding the ability to handle social functioning and bodily pain [16]. Although individuals with both mental and physical disease face the greatest health burdens, they are among the least studied [17]. Such burdens can be still greater for those of racial and ethnic minorities. Blacks and Hispanics experience larger declines in health than non-Hispanic Whites as they age, which is partly explained by their lower socioeconomic status reflecting adverse effects of racism, discrimination, and residential segregation; these may limit education and occupation, which contribute to poor living and working conditions throughout the life course [18,19]. Analyzing potential mediators for developing mental illnesses can help mitigate these disadvantages.

Health insurance coverage may be a particularly effective mediator of such risks. In the U.S., about 60% of adults between 19 and 64 years old have access to health insurance through their employer or a family member employer [20]. Prior to the passage of the Affordable Care Act (ACA), the uninsured (e.g., unemployed workers, and workers in the informal sector or “gig” economy), particularly older workers, had high premiums to access private health insurance [21]. The ACA expanded Medicaid to uninsured older adults whose income was no more than 138 percent of the federal poverty threshold [22]. It also subsidized private health insurance for some whose income was above 138 percent of the threshold without access to employer-sponsored health insurance [23].

For those older than 65, Medicare provides practically universal health care coverage [24]. Enrollment in Medicare’s private supplementary plan, Medicare Advantage has nearly doubled over the past decade [25], but such private plans can lead to inequities [26]. Medicare Advantage plan, also known as Medicare Part C, provides the same coverage as Medicare Part A (hospital insurance) and Part B (medical insurance), while offering additional benefits offered by private companies, such as coverage on vision, hearing, dental or benefits tailored to specific conditions. Medicare Advantage is associated with greater racial disparity regarding hospital readmissions than traditional Medicare is [27].

While recent U.S. policies have helped reduce the number of uninsured older adults, racial and ethnic minorities still have lower coverage rates, particularly at older ages before reaching age 65 or Medicare eligibility. Among U.S. individuals 19 to 64 years of age, 11.1 percent were uninsured in 2019, while among all individuals uninsured rates were higher among Blacks and Hispanics [28]. In prime working ages, Blacks and Hispanics are more likely than non-Hispanic Whites to have worked in low-skilled occupations and to have experienced disadvantages close to retirement age leading to involuntary job separation and lesser likelihood of reemployment [29,30]. Blacks and Hispanics are also more likely to work or supplement their income with a job that does not provide health insurance in the informal sector or in lower paid temporary or on-call “gig” economy jobs [31,32]. All this contributes to only 49.6 percent of Hispanics and 55.4 percent of Blacks reporting to have private health insurance, compared to 74.8 percent of non-Hispanic Whites [33].

Health insurance, whether public or private, increases healthcare utilization [24,34], which leads to better management of existing health conditions. Health insurance can relieve mental health risks by providing financial relief from the risk of high health costs [35,36]. This paper examines the association between private health insurance coverage and mental health among those with MCC in the United States by race and ethnicity. We examine the uninsured in comparison to those with private health insurance before age 65 and those with Medicare in comparison to Medicare and supplemental private insurance after age 65. Given prior research, this study hypothesizes that, for those with MCC, access to private health insurance (1) reduces the risk of developing depressive symptoms or cognitive impairment, (2) reduces such risk more for Blacks and Hispanics than for non-Hispanic Whites, and (3) reduces the risk of depressive symptoms more for adults in late middle age nearing entry into Medicare than for those who have reached the Medicare eligibility age of 65.

This study builds on previous research in several ways. First, to our knowledge, it is the first study to examine the relationship among private health insurance, mental health, and MCC. Second, it extends previous analyses to examine the context of race and ethnicity. Given that minority groups have lower rates of insurance coverage and higher risks of developing health conditions partly due to their lower socioeconomic status, it is crucial to examine the mediating effect of private insurance. Third, this study distinguishes older adults who have and have not reached the Medicare eligibility age of 65. Conducting separate analyses will provide additional insights on the benefits of extending insurance coverage to a younger cohort as well as on inequities among those who have reached Medicare eligibility.

## 2. Materials and Methods

### 2.1. Data Source

We used 12 waves of the Health and Retirement Study (HRS), a nationally representative, longitudinal study of older adults aged 50 and over in the United States. The biennial survey started in 1992 and contains a wide range of information, including variables on demographics, socioeconomic status, health and healthcare utilization, family, employment and retirement, and wealth. The HRS follows respondents until death and enrolls new cohorts as needed to maintain representation of all persons 50 or older. We use all waves through with data on depressive symptoms, with questions first introduced in 1994, and on cognitive tests, with questions first introduced in 1996.

### 2.2. Analytic Sample

We define chronic conditions as those for which the respondent reported a diagnosis including arthritis, diabetes, heart problem, hypertension, cancer, lung problem, and stroke. We compare those with two or more such conditions with those who have zero or one such condition. For respondents before age 65, we compare those with private healthcare access to those without, excluding those who are covered by Medicaid. For those 65 or older, we compare those with Medicare coverage to those with both Medicare and supplemental private insurance, excluding the less than 1 percent of respondents in this age group with no insurance. The small number of persons older than age 65 without health insurance may include older recent immigrants whose access to Medicaid and Medicare was reduced by the Personal Responsibility and Work Opportunity Reconciliation Act of 1996 [37]. We compare non-Hispanic Whites, non-Hispanic Blacks, and Hispanics.

For our final sample, we include all eligible observations with complete information. This results in a sample size of 71,323 person-year observations for Whites, 14,972 for Blacks, and 6428 for Hispanics. Our sample size for cognitive health is slightly smaller because the data is only available from 1996 rather than 1994 as for depressive symptoms.

### 2.3. Dependent Variable

We used the Center for Epidemiologic Studies Depression (CESD) scale to generate a binary indicator of depressive symptoms. The HRS contains a shorter version of the CESD with eight Yes/No questions measuring symptoms of depression and anxiety of the respondents. The scale ranges from 0 to 8, with a higher score indicating poorer mental health. Studies have validated that a CESD score of 4 and higher indicates symptoms of depression and anxiety [38,39]. We use this cutoff to indicate whether a respondent has depressive symptoms (1) or not (0).

We also used a binary variable to indicate cognitive impairment. Cognitive impairment in the HRS is based on a series of cognitive tests: immediate and delayed recall of 10 words, five trials of serial 7 s (i.e., having the respondent start at 100 and subtracting by 7 until reaching 65), and backward counting. From these tests, the HRS calculates a total score for cognitive impairment ranging from 0 to 27, with a score of 12 or greater defined as normal cognitive functioning, a score of 7 to 11 indicating as cognitive impairment but no dementia (CIND), and a score of 6 or less as dementia [40]. Our binary measure indicates whether an individual is in the CIND or dementia range (1) or has normal cognitive functioning (0). For respondents with missing variables, we derive proxy measures from the Cross-Wave Imputation of Cognitive Functioning Measures: 1992–2016 (Final, Version 6.0) [41].

### 2.4. Independent Variables

For our main independent variable, we used a binary variable indicating whether the respondent has access to private health insurance. HRS asks the number of plans purchased on the private market, including those purchased through an employer, individual insurance marketplaces, a union, or a trade association. For each wave, we created a variable which equals one for respondents with at least one private health insurance plan; for respondents without a private health insurance plan, this variable equals zero. As noted, for those age 50 to 64, we compare the uninsured to those with private health insurance, and for those age 65 and over, we compare individuals with Medicare to those with both Medicare and private insurance plans.

Covariates include time or wave dummies, age, gender, living arrangement (i.e., live alone, with a spouse/partner, or in an extended household) [42], and socioeconomic measures such as education levels and household income terciles [33,43].

### 2.5. Statistical Analysis

We show descriptive statistics stratified by race and ethnicity. We compare those with MCC to those without it. We then use longitudinal data methods to examine the association of health insurance status with the probability of depressive symptoms or cognitive impairment for non-Hispanic Whites, Blacks, and Hispanics. We conduct multivariate logistic regression with fixed-effects [44], using biennial longitudinal data for the same individuals from the mid-1990s to 2016. The advantages of using longitudinal data are a larger sample size which allows us to use econometrics methods to eliminate omitted variable bias for variables that do not change over time or that change uniformly over time for all individuals. Including individual fixed-effects allows us to control for unobserved time-invariant characteristics, including genetic characteristics that can affect mental health and the risk of chronic disease. Time or wave dummies allows us to control for time fixed effects [29,45]. For comparison purposes, we present estimation results for a sample with MCC and for one or without MCC. All models control for covariates. Standard errors are clustered at the individual level. We use Stata v14 (StataCorp LLC, College Station, TX, USA) for all analyses.

## 3. Results

Because we consider insurance in different ways for our sample based on respondent age, we generally split our analyses by age as well. For example, in our descriptive analysis, we first present results for persons 50 to 64 years of age, differentiating by whether they have private health insurance or no insurance. Recall that we do not consider persons in this age group who rely solely on public health insurance programs such as Medicaid. We then present results for persons at least 65 years of age, differentiating by whether they have a private insurance supplement to Medicare or rely solely on Medicare. Recall that we do not consider persons in this age group who do not qualify for Medicare. Likewise, for our tables on multivariate results, we first discuss those 50 to 64 years of age and then those at least 65 years of age. In our discussion, we note some themes that cut across both age groups.

### 3.1. Sample Characteristics

**Figure 1:**
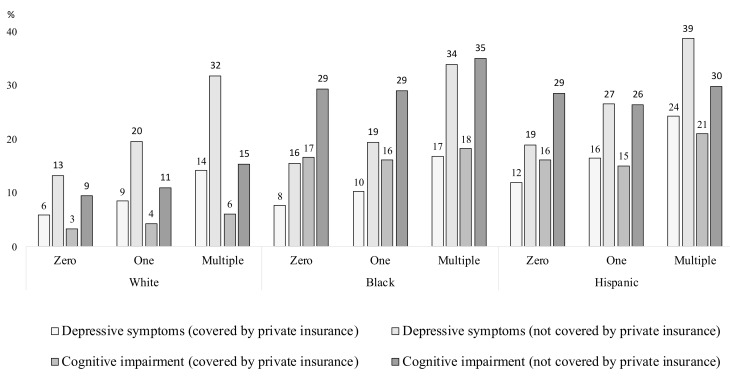
Mental conditions by private health insurance status, race/ethnicity, and number of chronic conditions for adults 50 to 64 years of age. *Notes*: The horizontal axis represents for non-Hispanic Whites, non-Hispanic Blacks, and Hispanics the number of chronic health conditions of the following: diabetes, heart problem, cancer, lung problem, hypertension, and stroke. Depressive symptoms are based on the Center for Epidemiologic Studies Depression Scale (CESD). which ranges from 0 to 8; a score of 4 or higher indicating symptoms of depression and anxiety [38,39]. Cognitive impairment is based on a cognitive score ranging from 0 to 27; a score of 12 or greater indicates normal cognitive functioning, a score of 7–11 as cognitive impairment no dementia (CIND), and a score of 6 or less as dementia [40]. For our purposes, a score less than 12 indicates cognitive impairment. Source: Health and Retirement Study (1994–2016).

Figure 1 shows the proportion of individuals 50 to 64 years of age by race or Hispanic origin, private insurance coverage, and number of chronic conditions who report depressive symptoms or cognitive impairments. For example, it indicates that 6% of Whites 50 to 64 years of age with private health insurance coverage and with no chronic conditions reported depressive symptoms. It also shows that 30% of Hispanics in that age group without private health insurance coverage and with multiple chronic conditions reported cognitive impairments.

Figure 1 shows that individuals 50 to 64 years old who do not have private health insurance, have multiple chronic conditions, and who are Black or Hispanic are more likely to have depressive symptoms or cognitive impairment. For all groups in Figure 1, and regardless of private health insurance status, depressive symptoms and cognitive impairments are greater for those with more chronic conditions. Depressive symptoms and cognitive impairments are greater for those without insurance than those with it. For Whites, depressive symptoms are more prevalent than cognitive impairments are. For Blacks, cognitive impairments are more prevalent than depressive symptoms. For Hispanics, cognitive impairments are more prevalent for those with no chronic conditions, but depressive symptoms are more prevalent for those with multiple chronic conditions. Depressive symptoms are greatest for Hispanics, while cognitive impairments are greatest for Blacks. Insurance does appear to mediate depressive symptoms; regardless of number of chronic conditions, depressive symptoms among Blacks and Hispanics with private insurance are less prevalent than they are among Whites without it.

**Figure 2:**
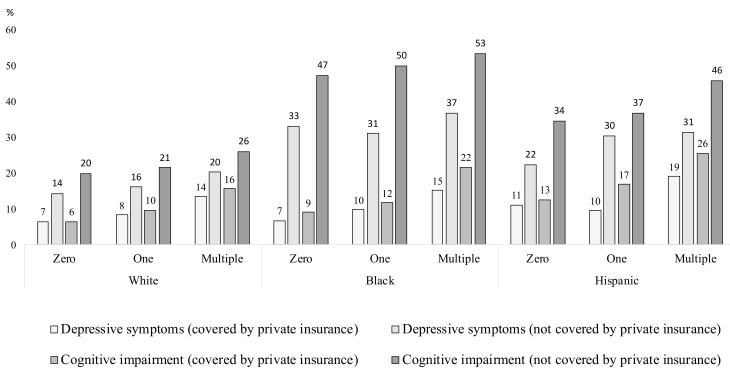
Mental conditions by private health insurance status, race/ethnicity, and number of chronic conditions for adults 65 years of age or older. *Notes*: The horizontal axis represents for non-Hispanic Whites, non-Hispanic Blacks, and Hispanics the number of chronic health conditions of the following: diabetes, heart problem, cancer, lung problem, hypertension, and stroke. Depressive symptoms are based on the Center for Epidemiologic Studies Depression Scale (CESD). Which ranges from 0 to 8; a score of 4 or higher indicating symptoms of depression and anxiety [38,39]. Cognitive impairment is based on a cognitive score ranging from 0 to 27; a score of 12 or greater indicates normal cognitive functioning, a score of 7–11 as cognitive impairment no dementia (CIND), and a score of 6 or less as dementia [40]. For our purposes, a score less than 12 indicates cognitive impairment. Source: Health and Retirement Study (1994–2016).

Figure 2 is identical to Figure 1 except it shows results for individuals who are at least 65 years of age and at least receive Medicare. Like Figure 1, it differentiates individuals by race or Hispanic origin, coverage by a private Medicare supplement, and number of chronic conditions. It shows the proportion of individuals in groups formed by these categories who report depressive symptoms or cognitive impairments. For example, it shows that 7% of Whites 65 years of age or older, with a private Medicare supplement, and with no chronic conditions report depressive symptoms. It also shows that 46% of Hispanics 65 or older covered only by Medicare and with multiple chronic conditions report cognitive impairments.

As Figure 1 shows, this figure also shows that individuals who are 65 years of age or older and do not have private health insurance, who have multiple chronic conditions, and who are Black and Hispanic are more likely to have depressive symptoms or cognitive impairment. Not surprisingly, cognitive impairments are generally greater for these groups than for the younger groups in Figure 1. Cognitive impairments here are also greater for Blacks than for Hispanics, for Hispanics than for Whites, for persons without a private insurance supplement than for those with one, and for persons with multiple chronic conditions. Depressive symptoms show many of these same patterns but tend to be greater for Hispanics than the other two groups. The difference in depressive symptoms between those with and without a private insurance supplement, however, is less pronounced among these groups than among the younger groups. For each of the nine groups defined by race, Hispanic origin, and number of chronic conditions, the rate of depressive symptoms for those without a private insurance supplement is no more than seven percentage points. In fact, for six of these groups, it is no more than two percentage points. Furthermore, the prevalence of depressive symptoms among all groups with Medicare coverage, and with or without a private supplement, is roughly comparable to those for the younger groups covered by private insurance. For example, among Whites with no chronic conditions, 6% of those 50–64 with private insurance, 7% of those with Medicare and a private supplement, and 6% of those with Medicare but no supplement report depressive symptoms. Similarly, among Hispanics with multiple chronic conditions, 24% of those 50–64 with private insurance, 19% of those with Medicare and a private supplement, and 26% of those with Medicare but no supplement report depressive symptoms. This suggests any insurance coverage may help minimize depressive symptoms.

Table 1 presents descriptive statistics for White, Black, and Hispanic respondents 50 to 64 years of age by their number of chronic conditions, here grouping together those with no chronic conditions or only one. We see that individuals with multiple chronic conditions have higher prevalence of depressive symptoms or cognitive impairment. Whites and Blacks with multiple chronic conditions are more likely to be uninsured than those without multiple conditions. Hispanics with and without multiple conditions have nearly identical rates of insurance coverage, albeit rates lower than those for Whites and Blacks. For Whites, depressive symptoms are more prevalent than cognitive difficulties regardless of the number of chronic conditions. For Blacks, cognitive difficulties are more prevalent. For Hispanics, depressive symptoms are more prevalent for those with multiple chronic conditions, while cognitive difficulties are more prevalent for others.

For Whites and Blacks, there is a gradient between education and health. That is, Whites and Blacks with multiple chronic conditions have, on average, about a half-year less of education than those without multiple conditions. There is no such gradient for Hispanics. For all three groups, there is a gradient between income and health: those with multiple chronic conditions have lower levels of income. Finally, while Blacks and Hispanics are more likely to live in extended households, among all three groups there is little relationship between living arrangements and the number of chronic conditions.

Table 2 is identical to Table 1 except it shows results for individuals who are at least 65 years of age. Like Table 1, it shows two groups of these individuals—those with multiple chronic conditions and those without—and differentiates by race and Hispanic origin as well. As in Table 1, Whites, Blacks, and Hispanics with multiple chronic conditions are more likely to have depressive symptoms or cognitive difficulties. Depressive symptoms are most prevalent for Hispanics, while cognitive difficulties are most prevalent for Blacks. While prevalence of dementia, not surprisingly, is higher for these groups of individuals, prevalence of depressive symptoms is lower, as one would surmise from Figure 1 and Figure 2 above. Whites are more likely than Blacks, and Blacks more likely than Hispanics, to have a private insurance supplement to Medicare. There is little difference in coverage, however, by number of chronic conditions.

There is also little to no education gradient between education and health. Whites with multiple chronic conditions have about 0.3 fewer years of education than other Whites. Nevertheless, there is virtually no difference in education by number of chronic conditions for Blacks and Hispanics. Similarly, while Blacks and Hispanics in this age group live in larger households, there is no little relationship between living arrangements and chronic conditions. Finally, there is virtually no relationship between income and number of chronic conditions. That is, the income structure for Whites, Blacks, and Hispanics, while differing from each other, does not differ by the number of chronic conditions individuals report.

In sum, we do find racial disparities on mental health outcomes that are unfavorable for Blacks and Hispanics, and particularly for those with MCC. There are also large disparities in access to private health insurance for Blacks and Hispanic adults. Furthermore, Blacks and Hispanics, particularly those with MCC, have lower levels of socioeconomic status than Whites do, exacerbating the disparities in accessing insurance. Nevertheless, when available, insurance may mitigate some of these disparities.

To better identify the association between private health insurance and mental health, we conducted multivariate analyses of the association between private health insurance and mental conditions by race ethnicity. We differentiated these analyses by age, race and Hispanic origin, and number of chronic conditions. These models show for those lacking a private insurance plan (50 to 64 years of age) or a private insurance supplement to Medicare (65 years of age or older) the change in risk for depressive symptoms or cognitive impairment. They control for age, education, living arrangements, household income, year of survey, and individual fixed-effects (e.g., family and genetic history). We turn next to these analyses.

### 3.2. Association between Private Health Insurance and Mental Health

We use a multivariate regression to estimate among those 50–64 the association of private health insurance with depressive symptoms and cognitive impairment by race and ethnic groups (Table 3). We also analyze for those 65 or older the association of private Medicare supplements with depressive symptoms and cognitive impairment by race and ethnic groups (Table 4). 

Panel A of Table 3 shows that being uninsured significantly increases risks of depressive symptoms for Whites, Blacks, and Hispanics 50 to 64 years of age with multiple chronic conditions. We find no association between cognitive impairment and lack of private health insurance for respondents in this group. This may be due to the small share of individuals 50 to 64 years of age with cognitive impairment. For those with MCC 50 to 64 years of age, 8% of Whites, 24% of Blacks, and 25% of Hispanics have cognitive impairment; for those with MCC and at least 65 years of age, 22% of Whites, 46% of Blacks, and 42% of Hispanics have cognitive impairment (see Table 1 and Table 2).

Panel B of Table 3 shows that, among those with no more than one chronic condition, being uninsured increases the risk of depressive symptoms for Blacks but not for Whites and Hispanics. It also shows a small increase in risk of cognitive impairment for Hispanics but no significant change in risk for Whites or Blacks.

Altogether, Table 3 indicates that, among the broader population, the mediating effect of private insurance on depressive symptoms is stronger for those with MCC than for those without it. At the same time, by focusing on racial differences, we can see that private health insurance also benefits the mental health of Blacks with good physical health.

Panel A of Table 4 shows the effects of private Medicare supplements on depressive symptoms and cognitive impairment among those at least 65 years of age with multiple chronic conditions. We find no difference in depressive symptoms between such individuals with and without a private supplement—supporting our earlier descriptive findings (Figure 2). We do, however, find that Blacks and non-Hispanic Whites with multiple chronic conditions but without a private Medicare supplement are more likely to have cognitive impairment.

Panel B of Table 4 shows the effects of Medicare supplements among those without multiple chronic conditions. We find no association between supplements and depressive symptoms for such individuals. We do, however, find that Blacks without a private Medicare supplement are more likely to have cognitive impairment. Our research suggests some possible association between private insurance supplements and cognitive impairment for Blacks, a mixed or modest association for Whites, but none for Hispanics. This suggests that other contributors to cognitive impairment should be analyzed. For sensitivity analysis, we conducted our analysis using the CESD and the reversed cognition scores instead of binary measures for the dependent variable, which produced overall similar estimates. The results are presented in a supplementary file (Table S1 and S2).

## 4. Discussion

Our study shows private insurance coverage is significantly associated with reduced risks of developing depressive symptoms for Whites, Blacks, and Hispanics 50 to 64 years of age with multiple chronic conditions. It also shows that a private Medicare supplement is significantly associated with reduced risks of cognitive impairment for Whites and Blacks 65 years of age or older with multiple chronic conditions. This is consistent with our first hypothesis, that private health insurance reduces the risk of developing mental health conditions.

We also find the mediating effect on depressive symptoms is strongly significant for adults in late middle age rather than for those with Medicare coverage. This supports our third hypothesis, that private insurance reduces the risk of depressive symptoms more for individuals in late middle age (i.e., before Medicare eligibility) than for those who have reached Medicare eligibility. We find insignificant effects of private health insurance on cognitive impairment for those 50 to 64 years of age, that could be mainly explained by the small proportion of individuals in this age group with cognitive impairment, as we discuss above. This is consistent with previous studies showing that cognitive impairment manifests more among individuals older than 78 years of age [13].

It is noteworthy that the relationship between insurance and mental health risks is stronger for Blacks than for Whites or Hispanics. This partially supports our second hypothesis, that private health insurance reduces the risk of developing a mental health condition more for Blacks and Hispanics than for Whites. In fact, for Blacks, the effects we found of private health insurance on mental health risks extend to those without multiple chronic conditions. While the effects on private health insurance for Hispanics are not as strong as they are for Blacks, other influences may affect Hispanics with multiple chronic conditions. For example, the small or insignificant estimates we found on private health insurance for cognitive health among Hispanics may be due to the fact that foreign-born, compared to native-born, tend to use less healthcare regardless of health coverage [46]. Such issues need further investigation. The effects we found from private insurance in reducing mental health risks for Whites with MCC, even if not as extensive as those for Blacks, indicate that the benefits of such insurance may be applicable to individuals of all racial/ethnic groups with multiple health conditions.

Although applicable to all groups, the positive health benefit of private health insurance could have greater impacts on minority race and ethnic groups, leading to increased quality of life and higher savings in direct and indirect health costs for them. Summary statistics show that Blacks and Hispanics have a higher prevalence of mental health conditions than Whites, regardless of coverage. This may be a result of lower socioeconomic status and poorer physical health, among other reasons. Previous research has documented larger declines in health for Blacks and Hispanics at older ages that reflect their health, social, and economic disadvantages throughout life [18]. Racial discrimination may further exacerbate mental health, affecting not only those directly experiencing it but also those with high levels of psychological distress who perceive it [47]. Furthermore, racial and ethnic minorities tend to use less healthcare and receive lower quality of care than nonminorities [48].

The ACA aimed to increase accessible healthcare to the uninsured by extending Medicaid coverage and providing subsidies for private health insurance. Following its full implementation in 2014 the number of the uninsured population below age 65 dropped to a historic low in 2016 of 26.7 million, however, it began to rise continuously to reach 28.9 million individuals being uninsured in 2019. This was an eight percent increase in the proportion of uninsured or a nine-percentage point increase of the uninsured rate from 10% in 2016 to 10.9% in 2019 [49]. Studies have shown that affordability is the biggest reason for having no coverage among the remaining uninsured [23,50]. Recent reports suggest the gap in coverage affordability has further widened in 2020 as millions have lost jobs and income from the ongoing COVID-19 pandemic [23]. Lack of health coverage will further threaten the health of those infected with the virus, especially for those with underlying chronic conditions, and aggravate disparities for minority groups.

Our study shows health coverage can mediate the risk of developing mental illnesses. Reducing the risk of mental health conditions through increased healthcare coverage can greatly reduce morbidity and healthcare costs. Patients with mental problems tend to use healthcare services for long periods of time, resulting in higher healthcare expenditures. Among high-cost patients, those who use mental health services spend more than others on overall healthcare [51]. Mental health problems also come with substantial indirect costs, such as productivity losses and caregiving costs [52]. The economic burden of dementia is substantial with the global burden expected to reach US$2 trillion by 2030 [53]. The significant influences we found on depressive symptoms among those 50 to 64 years of age without private health insurance implies extending coverage to those with MCC may help reduce long-term direct and indirect health costs.

Our study provides momentum for undertaking further steps to expand health insurance coverage, particularly targeting vulnerable groups. As of February 2021, 12 states have not adopted the Medicaid expansion as part of the ACA [54]. Some states which have not participated, such as Texas, has the highest rate of uninsured in the country [55]. Policy options could consider expanding subsidies or other affordable coverage options to targeted populations including long-term uninsured adults with multiple chronic health conditions. Among those with medical health conditions, long-term uninsured adults are much less likely to have received routine checkups and preventive services than insured adults [34]. Expanding healthcare access may reduce healthcare expenditures in the long-term, as discussed above.

Although private health insurance is related to lower likelihood of depressive symptoms and cognitive impairment, we do not know the underlying mechanism of this association. Increased coverage can lead to high healthcare utilization [24,34]. This, in turn, can increase treatment and improve management of the existing chronic conditions. This may further reduce the risk of developing mental health conditions. As previous research has shown, access to health care services and long-term care is relevant for those with dementia [56]. At the same time, the reduction in mental health risk may instead stem from the reduction in psychological distress associated with greater financial security [57]. The primary purpose of health insurance is to spread the risk of potential medical expenses, where individuals do not need to pay the full amount if substantial medical expenses occur [36]. The larger coefficients for Blacks suggest the possible risk spreading effect may be stronger for adults with lower socioeconomic status [58]. Future research should consider these issues.

There are several limitations of our study. First, we do not know the causal effect of private health insurance on mental health. In order to control for potential selection bias, we added covariates related to socioeconomic status, such as education levels and total household income, because persons with higher socioeconomic status may be more able to purchase private health insurance and be in better health. Individual fixed-effects also control for time-invariant variables omitted from the model, including genetic and family health history. Second, we cannot account for individuals who are undiagnosed. We expect some uninsured individuals are undiagnosed, even if they have a health condition. This suggests we may underestimate the proportion of uninsured adults with multiple chronic conditions. If this is the case, then the mediating effect of insurance may be even greater than what we found. Third, we only focused on the number of chronic conditions rather than the impact of each condition. The risk of developing a mental condition may be greater for specific diseases, with the different impact of each disease resulting in a different risk for developing a mental condition. Future research may investigate this further. Fourth we do not include those with access to Medicaid, which provides health insurance for older adults in poverty. While expansion of Medicaid since 2014 has helped reduce health disparities, Medicaid may provide lower quality of services than private health insurance, and its adult recipients may face additional barriers for receiving care [58,59,60]. Moreover, public insurance often provides limited protection compared to private [61,62]. Differences in coverage among insurance plans may also be an interesting factor to consider in future studies.

In spite of these limitations, our study provides several new insights for researchers and policymakers. By focusing on the mental and cognitive health of adults with MCC, we focused on those with the highest burdens but who have been least studied. Our study examines the association of private insurance on mental health, while most previous research on mental health focuses on public health insurances. Finally, by stratifying our sample by race and ethnicity, we provide additional policy implications for older minority populations.

## 5. Conclusions

This study shows that among older adults with MCC, private health insurance coverage is significantly associated with reduced risks of depressive symptoms for those nearing entry into Medicare and with cognitive impairment for adults age 65 and older. We find the relationship between private health insurance and reduced risk of mental health conditions is significant across all racial and ethnic groups but even stronger for Black adults. This implies the need to expand the accessibility and affordability of private health insurances to all groups, but particularly to minority populations.

## Figures and Tables

**Table 1:** Descriptive statistics by race/ethnicity and number of chronic conditions for persons 50 to 64 years of age.

Variables	Multiple Chronic Conditions			Zero or One Chronic Condition		
	White	Black	Hispanic	White	Black	Hispanic
*Mental Health*						
Depressive symptoms	17.65	23.14	30.79	8.45	11.72	17.95
Cognitive impairment	8.01	24.47	25.03	4.69	20.11	21.02
*Insurance coverage*						
Uninsured	19.75	36.85	44.98	12.47	28.71	44.87
Private insurance	80.25	63.15	55.02	87.53	71.29	55.13
*Demographics*						
Age (years)	58.85 (3.74)	58.37 (3.76)	58.17 (3.82)	57.46 (3.91)	56.93 (3.88)	56.92 (3.85)
Education (years)	13.07 (2.48)	12.41 (2.68)	10.09 (4.37)	13.67 (2.39)	12.97 (2.62)	10.02 (4.54)
Male	43.06	34.29	48.66	41.90	40.34	43.63
*Living arrangements*						
Live alone	14.68	23.01	9.92	13.39	21.45	8.48
Spouse/partner	52.22	25.95	29.20	52.33	26.59	26.74
Extended household	33.10	51.03	60.88	34.28	51.96	64.78
*Household income*						
Low	23.11	43.68	48.73	13.39	21.45	8.48
Medium	32.79	30.48	29.31	52.33	26.59	26.74
High	44.10	25.84	21.96	34.28	51.96	64.78
N, person-year	20,209	7156	3388	38,615	7953	6837
N, individuals	6946	2795	1397	11,585	3036	2520

Notes: Cells reflect percentages or mean values with standard errors in parentheses. Chronic conditions include a diagnosis of the following: arthritis, diabetes, heart problem, hypertension, cancer, lung problem, and stroke. Source: Health and Retirement Study (1994–2016).

**Table 2:** Descriptive statistics by race/ethnicity and number of chronic conditions for persons 65 years of age or older.

Variables	Multiple Chronic Conditions			Zero or One Chronic Condition		
	White	Black	Hispanic	White	Black	Hispanic
*Mental Health*						
Depressive symptoms	14.27	18.91	23.68	8.03	10.09	13.75
Cognitive impairment	22.33	46.38	41.75	17.40	41.78	33.54
*Insurance coverage*						
Medicare + Private insurance	64.42	42.63	28.82	68.88	43.60	30.87
Medicare	35.58	57.37	71.18	31.12	56.40	69.13
*Demographics*						
Age (years)	75.94 (7.10)	74.15 (6.82)	73.70 (6.48)	74.13 (6.90)	73.29 (6.86)	72.36 (6.28)
Education (years)	12.67 (2.67)	11.28 (3.35)	9.55 (4.31)	12.96 (2.73)	11.27 (3.59)	9.41 (4.42)
Male	43.16	37.01	42.17	41.08	41.20	46.02
*Living arrangements*						
Live alone	29.58	30.62	19.31	28.20	34.26	18.26
Spouse/partner	52.95	29.69	39.28	56.54	28.81	40.89
Extended household	17.47	39.69	41.41	15.27	36.93	40.85
*Household income*						
Low	37.93	58.30	61.22	35.39	57.58	58.84
Medium	37.97	27.80	28.45	36.35	27.77	29.25
High	24.10	13.89	10.33	28.26	14.65	11.91
N, person-year	51,114	7816	3040	30,392	3291	2284
N, individuals	12,781	2350	1028	8939	1171	787

Notes: Cells reflect percentages or mean values with standard errors in parentheses. Chronic conditions include a diagnosis of the following: arthritis, diabetes, heart problem, hypertension, cancer, lung problem, and stroke. Source: Health and Retirement Study (1994–2016).

**Table 3:** Association between private health insurance and mental conditions by race/ethnicity for individuals age 50 to 64.

	White	Black	Hispanic
Panel A. Sample with multiple chronic conditions			
Y = Depressive symptoms	0.40 *** [0.20, 0.59]	0.37 ** [0.11, 0.62]	0.35* [0.03, 0.68]
N, person-year	25174	5820	2801
Y = Cognitive impairment	0.20 [−0.07, 0.46]	0.23 [−0.04, 0.49]	−0.30 [−0.67, 0.08]
N, person-year	26,351	6756	2654
Panel B. Sample with zero or one chronic condition			
Y = Depressive symptoms	0.20 [−0.04, 0.43]	0.79 *** [0.41, 1.17]	0.05 [−0.24, 0.34]
N, person-year	7388	1735	2116
Y = Cognitive impairment	0.06 [−0.23, 0.35]	−0.10 [−0.41, 0.22]	0.33 * [0.02, 0.65]
N, person-year	3584	2000	1838

Notes: Cells in Y rows show coefficients for probability of being uninsured relative to having private insurance for each mental condition, with 95% confidence intervals in square brackets. All models control for age, education, living arrangements, household income, wave dummies, and individual fixed effects. * *p* < 0.05, ** *p* < 0.01, *** *p* < 0.001. Source: Health and Retirement Study (1994–2016).

**Table 4:** Association between private health insurance and mental conditions by race/ethnicity for individuals age 65 and over with Medicare coverage.

	White	Black	Hispanic
Panel A. Sample with multiple chronic conditions			
Y = Depressive symptoms	−0.02 [−0.13, 0.08]	−0.09 [−0.34, 0.15]	0.12 [−0.28, 0.52]
N, person-year	16191	2638	1176
Y = Cognitive impairment	0.15 ** [0.05, 0.24]	0.41 *** [0.20, 0.62]	0.20 [−0.18, 0.59]
N, person-year	21,027	3714	1290
Panel B. Sample with zero or one chronic condition			
Y = Depressive symptoms	−0.17 [−0.35, 0.00]	−0.04 [−0.53, 0.44]	0.61 [−0.03, 1.26]
N, person-year	6002	710	555
Y = Cognitive impairment	0.05 [−0.09, 0.19]	0.44 * [0.07, 0.82]	0.07 [−0.40, 0.55]
N, person-year	9302	1226	927

Notes: Cells in Y rows show the coefficients for probability of lacking a private supplement to Medicare coverage relative to having both Medicare and a private supplement for each mental condition; 95% confidence intervals are in square brackets. All models control for age, education, living arrangements, household income, wave dummies, and individual fixed effects. * *p* < 0.05, ** *p* < 0.01, *** *p* < 0.001. Source: Health and Retirement Study (1994–2016).

## Data Availability

Publicly available datasets were analyzed in this study. The data can be found here: https://hrs.isr.umich.edu/ (accessed on 15 February 2021).

## Supplementary Materials

The following are available online at https://www.mdpi.com/1660-4601/18/5/2615/s1, Table S1: Association between private health insurance and mental conditions by race/ethnicity for individuals age 50 to 64, Table S2: Association between private health insurance and mental conditions by race/ethnicity for individuals age 65 and over with Medicare coverage.
