# Supplementary material for: Private Insurance and Mental Health among Older Adults with Multiple Chronic Conditions: A Longitudinal Analysis by Race and Ethnicity
Source: Int J Environ Res Public Health. 2021 Mar 5;18(5):2615. doi: 10.3390/ijerph18052615 (PMC7967527; doi:10.3390/ijerph18052615)
Supplement: Supplementary file 1 [file ijerph-18-02615-s001.pdf]

Supplementary file

The results using the CESD and the reversed cognition score are shown as below. A higher score indicates worse health. The overall significance and direction of the coefficients remain similar to our findings presented in Table 3 and Table 4 where the dependent variable is a dummy variable indicating depressive symptoms and cognitive impairment.

Table 1. Association between private health insurance and mental conditions by race/ethnicity for individuals age 50 to 64.

	White	Black	Hispanic
Panel A. Sample with multiple chronic conditions			
Y = CESD (0–8)	0.19*** [0.08,0.30]	0.31*** [0.16,0.46]	0.08 [−0.17,0.33]
n, person-year	20208	7152	3387
Y = Cognition score (0–27)	0.04 [−0.17,0.25]	−0.17 [−0.47,0.12]	0.11 [−0.28,0.49]
n, person-year	18,322	6612	3235
Panel B. Sample with zero or one chronic condition			
Y = CESD (0–8)	0.09** [0.02,0.16]	0.24*** [0.12,0.37]	0.06 [−0.09,0.21]
n, person-year	38,608	7948	6837
Y = Cognition score (0–27)	0.04 [−0.12,0.19]	−0.14 [−0.44,0.16]	0.16 [−0.12,0.43]
n, person-year	33,759	7146	6309

Notes: Cells in Y rows show coefficients for probability of being uninsured relative to having private insurance for each mental condition, with 95% confidence intervals in square brackets. All models control for age, education, living arrangements, household income, wave dummies, and individual fixed effects. The cognition score is reversed so that a higher score indicates worse cognitive health. **p* < 0.05, ***p* < 0.01, ****p* < 0.001. *Source*: Health and Retirement Study (1994–2016).

Table S2. Association between private health insurance and mental conditions by race/ethnicity for individuals age 65 and over with Medicare coverage.

	White	Black	Hispanic
Panel A. Sample with multiple chronic conditions			
Y = CESD (0–8)	−0.00 [−0.04,0.03]	−0.07 [−0.17,0.03]	0.16 [−0.05,0.37]
n, person-year	51114	7814	3040
Y = Cognition score (0–27)	0.14*** [0.06,0.21]	0.32** [0.11,0.53]	0.15 [−0.21,0.51]
n, person-year	48,843	7447	2953
Panel B. Sample with zero or one chronic condition			
Y = CESD (0–8)	−0.03 [−0.07,0.01]	−0.11 [−0.25,0.03]	0.10 [−0.10,0.29]
n, person-year	30,390	3290	2284
Y = Cognition score (0–27)	0.14* [0.03,0.26]	0.03 [−0.34,0.40]	0.20 [−0.24,0.64]
n, person-year	26,773	2900	2114

Notes: Cells in Y rows show coefficients for probability of being uninsured relative to having private insurance for each mental condition, with 95% confidence intervals in square brackets. All models control for age, education, living arrangements, household income, wave dummies, and individual fixed effects. The cognition score is reversed so that a higher score indicates worse cognitive health. **p* < 0.05, ***p* < 0.01, ****p* < 0.001. *Source*: Health and Retirement Study (1994–2016).
